# Perioperative Electroacupuncture Can Accelerate the Recovery of Gastrointestinal Function in Cancer Patients Undergoing Pancreatectomy or Gastrectomy: A Randomized Controlled Trial

**DOI:** 10.1155/2021/5594263

**Published:** 2021-03-31

**Authors:** Guotong Qiu, Tao Huang, Yang Lu, Lipeng Zhang, Yajie Zhao, Yong Yuan, Hu Ren, Jun An, Jincao Zhou, Rongjun Li, Yongxing Du, Tuoran Wang, Peng Wang, Fang He, Yunqing Ding, Jianwei Zhang, Bin Han, Zhongmin Lan, Shulan Qi, Zongze Li, Jianyong Gao, Zongting Gu, Yuemin Sun, Xiaofeng Bai, Saderbieke Aimaiti, Yunmian Chu, Chengfeng Wang

**Affiliations:** ^1^State Key Lab of Molecular Oncology and Department of Pancreatic and Gastric Surgery, National Cancer Center, National Clinical Research Center for Cancer/Cancer Hospital, Chinese Academy of Medical Sciences and Peking Union Medical College, Beijing 100021, China; ^2^Department of Ophthalmology, Acupuncture and Moxibustion Hospital of China Academy of Chinese Medicine Science, Beijing 100700, China; ^3^Graduate School, Liaoning University of Traditional Chinese Medicine, Shenyang 110847, Liaoning, China; ^4^Donggaodi Community Health Service Station, Beijing 100076, China; ^5^Binzhou Hospital of Traditional Chinese Medicine, Binzhou 251800, Shandong, China

## Abstract

The effect of perioperative acupuncture on accelerating gastrointestinal function recovery has been reported in colorectal surgery and distal gastrectomy (Billroth-II). However, the evidence in pancreatectomy and other gastrectomy is still limited. A prospective, randomized controlled trial was conducted between May 2018 and August 2019. Consecutive patients undergoing pancreatectomy or gastrectomy in our hospital were randomly assigned to the electroacupuncture (EA) group and the control group. The patients in the EA group received transcutaneous EA on Bai-hui (GV20), Nei-guan (PC6), Tian-shu (ST25), and Zu-san-li (ST36) once a day in the afternoon, and the control group received sham EA. Primary outcomes were the time to first flatus and time to first defecation. In total, 461 patients were randomly assigned to the groups, and 385 were analyzed finally (EA group, *n* = 201; control group, *n* = 184). Time to first flatus (3.0 ± 0.7 vs 4.2 ± 1.0, *P* < 0.001) and first defecation (4.2 ± 0.9 vs 5.4 ± 1.2, *P* < 0.001) in the EA group were significantly shorter than those in the control group. Of patients undergoing pancreatectomy, those undergoing pancreaticoduodenectomy and intraoperative radiation therapy (IORT) surgery benefitted from EA in time to first flatus (*P* < 0.001) and first defecation (*P* < 0.001), while those undergoing distal pancreatectomy did not (*P*_flatus_=0.157, *P*_defecation_=0.007) completely. Of patients undergoing gastrectomy, those undergoing total gastrectomy and distal gastrectomy (Billroth-II) benefitted from EA (*P* < 0.001), as did those undergoing proximal gastrectomy (*P*=0.015). Patients undergoing distal gastrectomy (Billroth-I) benefitted from EA in time to first defecation (*P*=0.012) but not flatus (*P*=0.051). The time of parenteral nutrition, hospital stay, and time to first independent walk in the EA group were shorter than those in the control group. No severe EA complications were reported. EA was safe and effective in accelerating postoperative gastrointestinal function recovery. Patients undergoing pancreaticoduodenectomy, IORT surgery, total gastrectomy, proximal gastrectomy, or distal gastrectomy (Billroth-II) could benefit from EA. This trial is registered with NCT03291574.

## 1. Introduction

Pancreatic and gastric cancer are among the 10 most common cancers [1], and the incidence rate of pancreatic cancer has increased in recent years. Pancreatectomy and gastrectomy are by far the best treatments for these patients. Accordingly, postoperative gastrointestinal function is an important indicator in the recovery process. Postoperative ileus (POI) is usually the leading cause of abdomen discomfort and a prolonged hospitalization period. Accelerating the recovery of postoperative gastrointestinal function has become a key step in the recovery of abdominal surgery patients. Efforts to treat POI have included removing the stomach tube, putting the intestinal feeding tube, and getting out of bed early, but the effect has been limited.

Electroacupuncture was developed based on traditional Chinese medicine principles [2, 3]. Electroacupuncture at different acupoints has been used as a therapeutic intervention for many clinical issues. Ezzo et al. [4] found that electroacupuncture (EA) helped with chemotherapy-induced acute vomiting. Liu et al. [5] found that EA alleviated urinary leakage among women with stress urinary incontinence. Ng et al. [6] found that acupuncture reduced the duration of postoperative ileus after laparoscopic surgery for colorectal cancer. Chen et al. [7] found that transcutaneous electroacupuncture could accelerate bowel movement and alleviate POI after open gastrectomy. However, the experience with acupuncture of patients undergoing pancreatectomy and gastrectomy is limited, and the literature related to this question is rare. Moreover, there is no clinical study evaluating the effects of acupuncture before and after an operation. Therefore, we conducted a clinical trial to investigate whether acupuncture could accelerate the recovery of patients who underwent pancreatic or gastric surgery.

## 2. Methods

### 2.1. Study Design

This prospective, randomized, controlled trial was conducted at the Cancer Hospital Chinese Academy of Medical Sciences between May 2018 and August 2019. This study was conducted in accordance with the ethical principles of the Declaration of Helsinki (1964). Ethical approval was given by the clinical research ethics committee of the Cancer Hospital of the Chinese Academy of Medical Sciences, and written consent was obtained from all participants. Patients were informed about the possibility of withdrawing from the study at any time and for any reason without this decision affecting their treatment.

The treatment was performed in an independent space, and patients could not communicate with each other during the treatment. The acupuncturist team was composed of experienced practitioners with an average of 15 years of practice.

### 2.2. Participants

The patients were recruited from our hospital through posters. The pancreatic participants were suspected of having malignant tumors by CT or MR examination and would undergo pancreatectomy, including pancreaticoduodenectomy (PD), distal pancreatectomy (DP), and IORT surgery (patients were also treated with palliative bypass procedure). The gastric participants had histologically confirmed malignant tumors and would undergo gastrectomy, including local gastrectomy (LG), total gastrectomy (TG), proximal gastrectomy (PG), and distal gastrectomy (Billroth-I and Billroth-II) (DGB-I and B-II). Written informed consent was obtained prior to the study.

Inclusion criteria: participants who were 18–85 years of age and had no history of severe cardiovascular, liver, blood, or kidney diseases and no history of injury, infection, bruising, or bleeding at the acupoints.

Exclusion criteria: participants who had previously undergone abdominal surgery, chemotherapy, or radiation; had been diagnosed with other types of cancer; or had participated in similar studies.

### 2.3. Randomization

**Figure 1:**
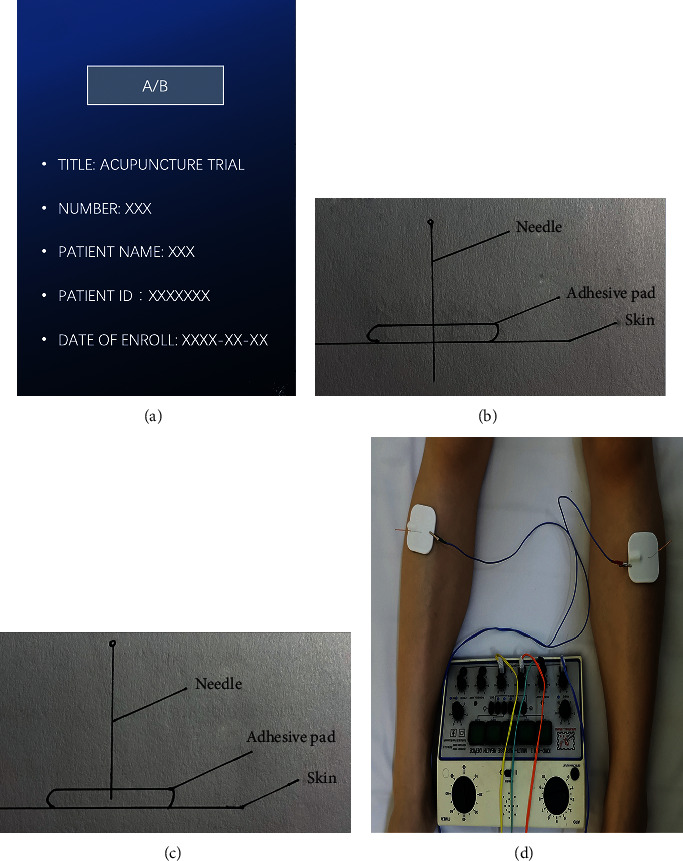
(a) Randomizing card. (b) Transcutaneous EA. (c) Sham EA with adhesive pads. (d) EA in patients.

The patients were randomly assigned into two groups, the EA group (patients who received preoperative and postoperative transcutaneous EA) and the control group (patients who received no transcutaneous EA), with a randomizing card (Figure 1). The acupuncturist randomly selected the card, and the patient's group was defined by scraping away the coating to reveal A or B. Outcome evaluators and statisticians were blind to EA group location.

To maximize the blindness of the participants, a placebo needle and a fake electric needle design were used (Figure 1) in the control group, which used adhesive pads and blunt placebo needles that were similar in appearance to traditional needles but did not penetrate the skin. The design of the fake electric needle included a connecting wire with an internal disconnection and made the same clicking sound as the EA needle, yet without any current output.

### 2.4. Interventions

We used hand needling combined with electroacupuncture. Our acupuncturist team was composed of experienced practitioners with an average of 15 years of practice (15.1 ± 8.58 years). We selected sterile acupuncture needles of Hwato brand (Suzhou Medical Supply Factory Co., Ltd.) for acupuncture, with specifications of 0.22 × 25 mm and 0.25 × 40 mm, respectively. The EA instrument was the KWD808-I (produced by Changzhou Wu-jin Medical Instrument Co, Ltd). The electroacupuncture parameters were alternating mode with 2–50 Hz and 2 mA. According to our clinical routine electroacupuncture experience, and the time of electroacupuncture that patients can tolerate, the electroacupuncture stimulation time was approximately 20 minutes once a day at 15:00.

**Figure 2:**
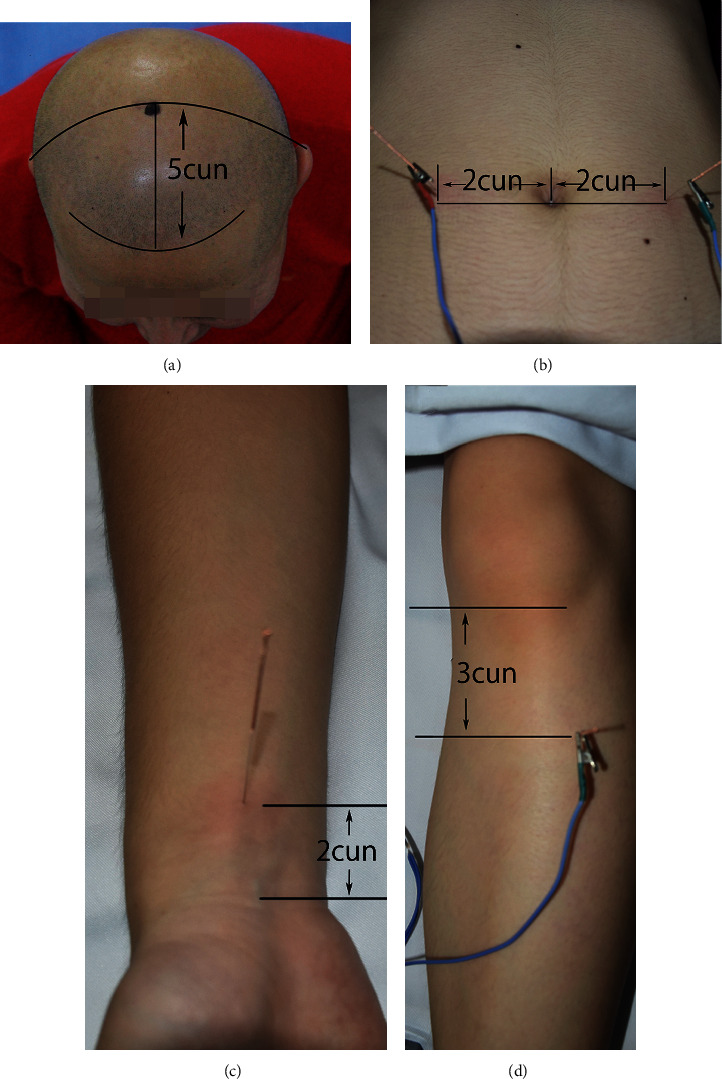
(a) Bai-hui (GV 20). (b) Tian-shu (ST25). (c) Nei-guan (PC6). (d) Zu-san-li (ST36).

The EA group was given electroacupuncture before and after surgery. The control group was given sham electroacupuncture (same time and frequency). Participants in the EA group received stimulation at Bai-hui (GV20, located on the top of the head, see Figure 2(a)) and the unilateral Nei-guan (PC6, located on 2 cun above the transverse crease of the wrist; 1 cun is approximately 3.33 cm, see Figure 2(c)) with needles of 0.22 × 25 mm; the bilateral Tian-shu (ST25, located on the middle abdomen, 2 cun lateral to the center of the umbilic, see Figure 2(b)) and the bilateral Zu-san-li (ST36, located on the anterolateral side of the leg, 3 cun bellow Dubi (ST35), one finger breadth from the anterior crest of the tibia 0.5 cun lateral to the extremity of the coccyx, see Figure 2(d)) with needles of 0.25 × 40 mm.

In the EA group, after skin disinfection, acupuncture needles were inserted through the adhesive pad into Bai-hui, Nei-guan, Tian-shu, and Zu-san-li, and then the needles were inserted perpendicularly through adhesive pads approximately 20–30 mm into the skin. Following needle insertion, small, equal manipulations of twirling, lifting, and thrusting were performed on all needles to reach de qi (a composite of sensations including soreness, numbness, distention, heaviness, and other sensations), which is believed to be an essential component of acupuncture efficacy. Paired electrodes from the electroacupuncture apparatus were attached transversely to the needle.

### 2.5. Main Outcome Measures

The primary outcomes of our study were the time (in days) from the end of the operation to the first observation of flatus and defecation. Secondary outcomes were the time of first independent walk, hospital stay, gastrointestinal function score which was evaluated at admission and the day after surgery (Supplemental 1), and EA complications. The data were recorded and measured by an independent research assistant.

### 2.6. Statistical Analysis

Descriptive analysis was used to summarize the characteristics and surgical results with the mean (standard deviation) of quantitative variables and the frequency (percentage) of qualitative variables. Student's *t*-test is used for continuous variable data and contingency table test is used for classified variable data. Compare baseline classification data with Pearson chi square test or Fisher exact test. Multiple linear regression analysis was used to determine independent prognostic indicators. All examinations were bilateral; *P* < 0.05 was significant. IBM SPSS statistical version 26.0.

## 3. Results

### 3.1. Main Electroacupuncture Outcomes

**Figure 3:**
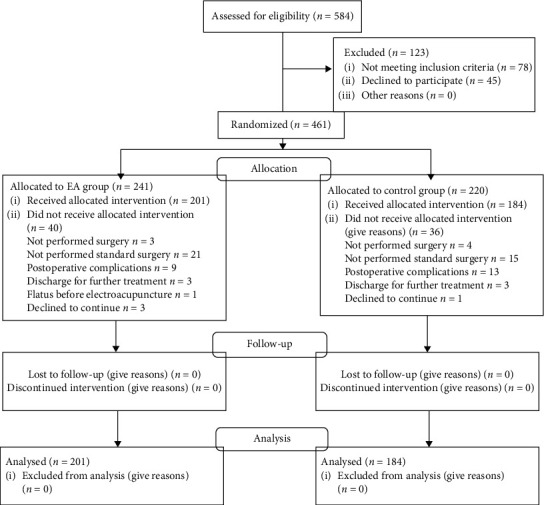
The CONSORT diagram.

Of the 584 patients who were eligible for the study, 461 patients who were randomized to the EA or control group. Finally, 385 were analyzed in the study. The CONSORT diagram details the flow chart (Figure 3). The study groups were comparable in baseline and operation characteristics (Table 1).

The primary outcome is compared in Table 2. The data is approximately normal distribution (Histogram and normal distribution curve are shown in Supplemental 2 and 3; the kurtosis and skewness values are shown in Supplemental 4). Compared with the control group, the EA group had significantly shorter times to first flatus (3.0 ± 0.7 vs 4.2 ± 1.0 *P* < 0.001) and first defecation (4.2 ± 0.9 vs 5.4 ± 1.2, *P* < 0.001). We further compared patients according to surgery type. Among those undergoing open surgery, the times to first flatus and defecation in the EA group were significantly shorter than those in the control group (flatus 3.1 ± 0.7 vs 4.2 ± 1.1, *P* < 0.001; defecation 4.3 ± 0.9 vs 5.5 ± 1.2, *P* < 0.001). The same pattern was found in laparoscopic surgery (flatus 2.8 ± 0.7 vs 3.9 ± 0.9, *P* < 0.001, defecation 3.9 ± 0.9 vs 5.1 ± 1.1, *P* < 0.001).

For the surgery types in pancreatectomy, including pancreatoduodenectomy (PD) and IORT surgery, patients could benefit from EA treatment (PD: flatus 3.4 ± 0.7 vs 5.3 ± 1.1, defecation 5.0 ± 0.7 vs 7.0 ± 1.2; IORT flatus 3.1 ± 0.6 vs 4.2 ± 0.8, defecation 4.3 ± 0.7 vs 5.5 ± 1.0, *P* < 0.001). However, patients undergoing distal pancreatectomy did not benefit from EA in time to flatus (2.9 ± 0.6 vs 3.4 ± 1.0, *P*=0.157) or defecation (3.7 ± 0.6 vs 4.4 ± 0.7, *P*=0.007) completely.

Patients undergoing gastrectomy (flatus defecation), including total gastrectomy (flatus 3.0 ± 0.6 vs 4.5 ± 0.8, defecation 4.3 ± 0.7 vs 5.8 ± 1.0, *P* < 0.001), proximal gastrectomy (flatus 3.1 ± 0.7 vs 3.8 ± 1.0, *P*=0.015, defecation 4.2 ± 0.8 vs 5.2 ± 1.4, *P*=0.015), and distal gastrectomy (B-II) (flatus 3.0 ± 0.7 vs 4.6 ± 0.9, defecation 4.1 ± 0.9 vs 5.3 ± 0.9, *P* < 0.001), could benefit from EA. However, patients undergoing local gastrectomy (flatus 2.0 ± 1.0 vs 3.2 ± 0.8, *P*=0.116; defecation 3.3 ± 1.2 vs 3.8 ± 0.8, *P*=0.528) or distal gastrectomy (Billroth-I) did not benefit from EA completely (flatus 2.8 ± 0.9 vs 3.7 ± 1.2; *P*=0.051, defecation 3.6 ± 1.0 vs 4.9 ± 1.4, *P*=0.012).

### 3.2. Secondary Outcomes

The first time to walk independently (3.7 ± 1.0 vs 4.0 ± 1.00, *P*=0.026) and hospital discharge (10.6 ± 5.4 vs 13.1 ± 6.7, *P* < 0.001) (Table 3) in the EA group were significantly shorter than those in the control group. The postoperative gastrointestinal function score (Supplement 1), including flatus, defecation, gastric tube drainage, intestinal feeding tube, borborygmus, diet, stomachache, nausea, and vomiting, was evaluated on postoperative days 1, 3, 5, and 7. On the first day after surgery, it was comparable in both groups. However, on days 3, 5, and 7, the gastrointestinal function score in the EA group was obviously higher than that in the control group (*P*_3_ < 0.001, *P*_5_=0.001, *P*_7_ < 0.001). The time to starting a soft diet in the EA group was earlier than that in the control group (4.7 ± 1.7 vs 6.0 ± 2.0, *P* < 0.001) (Table 3).

Many confounding factors (age, sex, body mass index, smoking, drinking, laparoscopy or not, and intraoperative blood transfusion) could have affected gastrointestinal motility and the duration of hospital stay. Their effects were analyzed by multiple linear regression (Table 4). The use of EA was an independent predictor of shorter time to flatus (regression coefficient = −0.535; 95% confidence interval [−1.288,−0.934]; *P* < 0.001), shorter time to defecation (regression coefficient = −0.51; 95% confidence interval [−1.425, −1.022]; *P* < 0.01), shorter time to independent walk (regression coefficient = −0.169; 95% confidence interval [−0.511, −0.176]; *P* < 0.001), and shorter hospital stay (regression coefficient = −0.21; 95% confidence interval [−3.722, −1.456]; *P* < 0.001).

### 3.3. Safety and Complications

Of the 385 patients analyzed, 1 patient complained about dizziness, and no skin hemorrhage or skin infection was reported. As for surgical complications, including death, postoperative bleeding, anastomotic leakage, and abdominal infection, there was no significant difference between groups (*P* > 0.05). In the EA group, the occurrence of gastroparesis syndrome was less than that in the control group (*P*=0.037), indicating the superiority of EA.

## 4. Discussion

POI is the most common iatrogenic complication after abdominal surgery. Its symptoms include abdominal pain, abdominal distension, nausea, vomiting, decreased or disappeared bowel sounds, delayed recovery in terms of time to flatus and time to defecation, and difficulty passing stools or tolerating a solid diet, causing severe complications such as wound dehiscence or pulmonary complications. It will also increase patients' hospital stay and medical cost. In a retrospective cohort study from over 500 hospitals in the USA, ileus was found to be an important predictor of increased postoperative hospital stay and cost in patients undergoing colectomy [8]. The economic impact of POI has been estimated at $750 million per year in the US [9, 10]. The pathophysiology to POI is multifactorial. In a review about POI, the activation of a local inflammatory response within the intestinal muscularis externa was an accepted pathophysiological mechanism [11]. A pilot study conducted by The et al. [12] found that Ketotifen can improve gastric emptying after abdominal surgery, indicating mast cell stabilizers were a putative therapy for POI.

Modern medicine to this problem was limited while Chinese medicine has accumulated much experience in POI. Acupuncture has been used as a minimally invasive and comprehensive treatment for gastrointestinal motility disorders in China for thousands of years. Chen et al. [7] found that transcutaneous electroacupuncture accelerated bowel movements. EA has also been widely accepted by Western clinicians and patients and has become an effective method for the treatment of various gastrointestinal conditions. In addition, acupuncture can relieve several symptoms, including pain, stroke, erectile dysfunction, nausea and vomiting, depression, gastric motility, and obesity. Ulloa et al. [13] also found that EA could help to control immune and organ functions. However, the use of acupuncture in the treatment of postoperative intestinal obstruction has not been extensively investigated, especially after pancreatectomy, and high-quality data related to this very clinically relevant question in Chinese and Western literature are scarce.

### 4.1. Primary Outcome

We report the first prospective randomized clinical trial (RCT) evaluating the effectiveness of EA in patients undergoing pancreatectomy. The effectiveness of EA treatment in other abdominal operations has been reported, including colorectal surgery and gastrectomy. However, the curative effect after pancreatectomy, especially in pancreaticoduodenectomy, the most complicated abdominal surgery, remains to be proven. In our study, we expanded the sample size and classified the surgery type into PD, IORT surgery, distal pancreatectomy, local gastrectomy, proximal gastrectomy, total gastrectomy, and distal gastrectomy (Billroth-I and II).

Pancreatectomy, especially pancreaticoduodenectomy, is known for its complicated and difficult recovery. In a multicenter RCT conducted by Perinel et al. [14], there was no significant difference in the postoperative time to first flatus between the nasojejunal early enteral nutrition group and total parenteral nutrition group, whose average times were 6.7 and 5.5 days (*P*=0.767). In our study, the postoperative time to first flatus was (3.4 ± 0.7 and 5.3 ± 1.1) days, and the time to first defecation was (5.0 ± 0.7 vs 7.0 ± 1.2) days. We obviously improved the postoperative gastrointestinal recovery. The improvement was 1.9 days for flatus and 2 days for defecation, which could markedly improve the postoperative quality of life. The same result was found in IORT surgery (including palliative bypass procedure), which is used to treat head pancreatic adenocarcinoma. However, a positive result was not found in distal pancreatectomy, either in time to first flatus (2.9 ± 0.6 vs 3.4 ± 1.0, *P*=0.45) or in time to first defecation (3.7 ± 0.6 vs 4.4 ± 0.7, *P*=0.057). Comparing this result with other positive surgery type groups, we found that the average times to first flatus and defecation in the DP control group were closer to those of the EA patients of all surgery types except local gastrectomy, and this last group did not benefit from EA treatment (flatus 2.0 ± 1.0 vs 3.2 ± 0.8, *P*=0.126; defecation 3.3 ± 1.2 vs 3.8 ± 0.8, *P*=0.576).

The effect of EA in a previous study on gastrectomy was limited. Chen et al. [7] found that transcutaneous electroacupuncture accelerated bowel movements in patients undergoing open gastrectomy but laparoscopic surgery. Meng et al. [15] conducted a study in 90 patients with prolonged POI who were randomly selected and given electroacupuncture or no acupuncture after the operation. No significant differences between the two groups were reported. In our study, we expanded the sample size to 265, including gastric cancer and gastric stromal tumor patients receiving TG, PG, DG, and LG, and performed EA preoperatively and postoperatively. Patients undergoing TG, PG, or DGB-II benefitted from EA, whether open or laparoscopic. Patients undergoing DGB-I benefitted from EA in defecation (3.6 ± 1.0 vs 4.9 ± 1.4, *P*=0.017) but not flatus (2.8 ± 0.9 vs 3.7 ± 1.2, *P*=0.101). Patients undergoing LG did not benefit from EA in either outcome (*P*_flatus_=0.126, *P*_defecation_=0.576). In the RCT conducted by Lee et al. [16], laparoscopic distal gastrectomy shows benefits in faster recovery for locally advanced gastric cancer. Their average postoperative time to flatus was shortened from 3.7 to 3.5 days (*P*=0.025). In our study, the time went from 4.0 to 2.9 days. The effect of EA is obvious.

According to the principles of traditional Chinese medicine, EA can stimulate the meridian points to release endorphins in the central nervous system and regulate the physiological response. Hui Ouyang et al. also found that the EA at PC6 and ST36 could probably enhance vagal activity [2]. Iwa et al. [17] found that EA at ST-36 stimulates glutaminergic neurons in the brainstem resulting in improvement of stress-induced delay of gastric emptying via central GABA (A) and GABA (B) receptors. Chen et al. [18] found that EA is able to restore RD-induced impairment in antral motility and GSW by possibly enhancing vagal activity and is partially mediated via the opioid pathway.

Combined with our results, we recommend that patients undergoing pancreatectomy or gastrectomy, including PD, IORT surgery, TG, PG, and DGB-II, receive preoperative and postoperative EA treatment.

### 4.2. Secondary Outcome

The time to independent walking in the EA group was earlier than that in the control group (3.7 ± 1.0 vs 4.0 ± 1.00, *P*=0.005). During postoperative recovery, earlier walking independently resulted in faster gastrointestinal function recovery. In addition, less parenteral nutrition was needed. Finally, the patients were discharged earlier. In our study, the hospital stay was approximately 3 days less in the EA group (10.6 ± 5.3 vs 13.1 ± 6.7, *P* < 0.001). Objectively, the basis of fast recovery is stable and excellent surgery procedure. Besides, gastrointestinal function score, which included several criteria for evaluating gastrointestinal symptoms, was significantly higher postoperatively compared with preoperatively.

### 4.3. Electroacupuncture Method

In this study, Bai-hui (GV20), Nei-guan (PC6), Tian-shu (ST25), and Zu-san-li (ST36) were used. Xu et al. found that EA at GV20 has a protective effect on sleep deprivation-induced depression-like behavior and cognitive impairment, which indicates humans can benefit from EA in sleep [19]. Lu et al. found that EA at ST36 regulate gastric motility via vagovagal and sympathetic reflexes mediated through M2/3 and *β*1/2 receptors, respectively [20]. Li et al. found that both EA and moxibustion at ST25 can lower the pressure of gastric, and the effect of EA is better than that of moxibustion in normal rats [21]. Lu et al. found that EA at PC6 could promote efferent vagus nerve activity and increase gastric motility [22]. Based on the above research and the clinical work experience of our acupuncturists, we selected these four acupoints as the stimulation points. Electroacupuncture was performed at 2 Hz, as this frequency has been demonstrated to reduce the symptoms of dyspepsia in diabetic gastroparesis patients, promote solid gastric emptying, and enhance the regularity of gastric myoelectric activity in healthy people [23–26]. We hypothesized that the combination of various therapeutic effects of electroacupuncture at these acupoints would achieve encouraging and beneficial results for our patients. Finally, the patients in our study obtained positive results from this kind of EA treatment.

### 4.4. Postoperative Complications

Of the 385 patients who were analyzed, 1 patient complained about dizziness, and no skin hemorrhage or skin infection was reported. Regarding surgical complications, there was no statistical significance in the rate of death, postoperative bleeding, anastomotic leakage, or abdominal infection between the two groups (*P* > 0.05). However, in the EA group, gastroparesis syndrome was less common than in the control group (*P*=0.013), which indicates the positive effect of EA in accelerating bowel movement.

In contrast to other enhanced rehabilitation methods, EA programs require only a trained acupuncturist. EA is easier to implement and far less labor-intensive than the complex elements of other fast-track projects, yet it can bring the same benefits to patients in terms of faster postoperative recovery and shorter hospital stay.

## 5. Conclusions

Our study has shown that perioperative EA in patients undergoing pancreatectomy or gastrectomy was safe and efficient. EA was an effective measure to accelerate postoperative gastrointestinal function recovery. Patients undergoing PD, IORT surgery, TG, PG, or DGB-II could benefit from preoperative and postoperative EA treatment. EA can shorten parental nutrition maintenance, accelerate walking independently, and decrease hospital stay.

### 5.1. Strengths and Limitations

The current study was strengthened by (a) the aid of a physician licensed in traditional Chinese medicine in locating the acupoint, (b) a separate outcome assessor and intervener to prevent measurement bias, and (c) preoperative and postoperative intervention to discover the full scope of the technique. Our study had several limitations. First, the study population represented a highly selected group of patients who underwent uncomplicated elective resection of gastric or pancreatic cancer. Patients with benign lesions were not recruited. Patients with colorectal or liver cancer were not included. Therefore, we cannot determine what effect this technique will have on other patients undergoing abdominal surgery. Future studies should target a more diversified population. More complicated patients are apparently more likely to develop prolonged ileus and morbidity after surgery, and it is also uncertain whether EA will be beneficial to them. Second, we did not use a fast-track perioperative program because it was not the standard of care in our institution. The possible combined effects of EA and the fast-track program on the clinical outcomes after gastric and pancreatic surgery will be an important area for further research. Further studies are needed to address this issue.

## Figures and Tables

**Table 1:** Baseline characteristics.

Variables	EA group (*n* = 201)	Control group (*n* = 184)	*P* value
Sex (M : F)	121 : 80	115 : 69	0.643
Age, yrs.	57.0 ± 10.8	56.7 ± 10.3	0.334
BMI, kg/m^2^	24.1 ± 4.0	24.1 ± 3.1	0.946
Diabetes (%)	32 (15.9)	30 (16.3)	0.918
Smoking (%)	61 (30.3)	70 (38)	0.111
Drinking (%)	57 (28.4)	64 (34.8)	0.175

ASA score (%)			0.783
I	68 (33.8)	57 (31)	
II	112 (55.7)	109 (59.2)	
III	21 (10.4)	18 (9.8)	
Preoperative gastrointestinal function score	103.9 ± 12.2	102.3 ± 12.5	0.199
Diagnosis			0.477
Gastric cancer	137	128	
Pancreatic adenocarcinoma	57	44	
Carcinoma of bile duct	0	1	
Periampullary adenocarcinoma	1	3	
Duodenum carcinoma	3	5	
Gastric stromal tumor	1	3	
Cystic and solid tumor of the pancreas	2	0	

Laparoscope (%)	52 (25.9)	38 (20.7)	0.227
Kind of surgery			0.768
Local gastrectomy	3	5	
Proximal gastrectomy	16	18	
Total gastrectomy	36	26	
Distal gastrectomy(Billroth-I)	13	13	
Distal gastrectomy(Billroth-II)	69	69	
Pancreaticoduodenectomy	25	17	
IORT surgery	26	20	
Distal pancreatectomy	13	16	

Operation time (min)	207.5 ± 64.5	195.5 ± 57.5	0.055
Intraoperative blood loss (ml)	147.5 ± 303.0	142.5 ± 261.0	0.863
Intraoperative blood transfusion (ml)	106.5 ± 352.9	77.7 ± 261.1	0.368

BMI: body mass index; ASA: American Society of Anesthesiologists; IORT: intraoperative radiation therapy.

**Table 2:** EA outcome.

Variable (d, mean, SD)	Time to first flatus (day)			Time to first defecation (day)		
	EA group (*n* = 201)	Control group (*n* = 184)	*P* value	EA group (*n* = 201)	Control group (*n* = 184)	*P* value
Total	3.0 ± 0.7	4.1 ± 1.0	<0.001	4.2 ± 0.9	5.4 ± 1.2	<0.001
Op surg	3.1 ± 0.7	4.2 ± 1.1	<0.001	4.3 ± 0.9	5.5 ± 1.2	<0.001
La surg	2.8 ± 0.7	3.9 ± 0.9	<0.001	3.9 ± 0.9	5.1 ± 1.1	<0.001
LG	2.0 ± 1.0	3.2 ± 0.8	0.116	3.3 ± 1.2	3.8 ± 0.8	0.528
PG	3.1 ± 0.7	3.8 ± 1.0	0.015	4.2 ± 0.8	5.2 ± 1.4	0.015
TG	3.0 ± 0.6	4.5 ± 0.8	<0.001	4.3 ± 0.7	5.8 ± 1.0	<0.001
DG(B–I)	2.9 ± 0.9	3.7 ± 1.2	0.051	3.6 ± 1.0	4.9 ± 1.4	0.012
DG (B-II)	3.0 ± 0.7	4.6 ± 0.9	<0.001	4.1 ± 0.9	5.3 ± 0.9	<0.001
PD	3.4 ± 0.7	5.3 ± 1.1	<0.001	5.0 ± 0.7	7.0 ± 1.2	<0.001
IORT	3.1 ± 0.6	4.2 ± 0.8	<0.001	4.4 ± 0.7	5.5 ± 0.9	<0.001
DP	2.9 ± 0.6	3.4 ± 1.0	0.157	3.7 ± 0.6	4.4 ± 0.7	0.007

EA: electroacupuncture; Op: open; La: laparoscopy; LG: local gastrectomy; PG: proximal gastrectomy; TG: total gastrectomy; DG: distal gastrectomy; PD: pancreaticoduodenectomy; IORT: intraoperative radiation therapy; DP: distal pancreatectomy

**Table 3:** Postoperative characteristics.

Variables	EA group (*n* = 201)	Control group (*n* = 184)	*P* value
Gastrointestinal function score			
Postoperative day 1	53.0 ± 7.9	52.7 ± 7.1	0.651
Postoperative day 3	65.2 ± 11.0	61.5 ± 10.9	0.001
Postoperative day 5	84.5 ± 10.1	78.9 ± 11.1	<0.001
Postoperative day 7	96.0 ± 7.2	91.9 ± 7.2	<0.001

Start of soft diet (day)	4.7 ± 1.7	6.0 ± 2.0	<0.001
Walk independently (day)	3.7 ± 1.0	4.0 ± 1.00	0.005
Hospital stay (day)	10.6 ± 5.3	13.1 ± 6.7	<0.001
Postoperative complications			
Death	0	0	
Postoperative bleeding	0	1	0.319
Anastomotic leakage	4	4	0.9
Gastroparesis syndrome	3	10	0.037
Abdominal infection	5	3	0.557
Lung infection	6	5	0.875
Urinary tract infection	2	3	0.583

Acupuncture complications			
Skin hemorrhage	0	0	
Skin infection	0	0	
Dizziness	1	0	

EA: electroacupuncture.

**Table 4:** Multiple linear regression analysis.

Variable	Coefficient B	*t*	95% CI for B	*P* value
Independent predictors of time to flatus				
EA (yes = 1, no = 0)	−0.535	−12.343	[−1.288, −0.934]	<0.001
Laparoscopic (yes = 1, no = 0)	−0.121	−2.753	[−0.507, −0.084]	0.006
Operation time (min)	0.158	3.511	[0.001, 0.004]	0.001

Independent predictors of time to defecation				
EA (yes = 1, no = 0)	−0.51	−11.951	[−1.425, −1.022]	<0.001
Laparoscopic (yes = 1, no = 0)	−0.167	−3.851	[−0.699, −0.212]	<0.001
Sex (male = 1, female = 0)	−0.102	−2.134	[−0.480, −0.020]	0.033
Operation time (min)	0.245	5.516	[0.003, 0.006]	<0.001

Independent predictors of independent walk				
EA (yes = 1, no = 0)	−0.169	−4.028	[−0.511, −0.176]	<0.001
Laparoscopic (yes = 1, no = 0)	−0.197	−4.665	[−0.674, −0.274]	<0.001
Sex (male = 1, female = 0)	−0.12	−2.58	[−0.443, −0.060]	0.01
Age	0.337	8.011	[0.025, 0.041]	<0.001
Operation time (min)	0.294	6.571	[0.003, 0.006]	<0.001

Independent predictors of hospital stay				
EA (yes = 1, no = 0)	−0.21	−4.493	[−3.722, −1.456]	<0.001
Laparoscopic (yes = 1, no = 0)	−0.171	−3.615	[−3.838, −1.134]	<0.001
Operation time (min)	0.264	5.428	[0.017, 0.036]	<0.001
Intraoperative blood transfusion (yes = 1, no = 0)	0.15	1.875	[0.0139, 5.840]	0.062

EA: electroacupuncture.

## Data Availability

Data are available upon request to the corresponding author.

## Abbreviations

EA: Electroacupuncture
IORT: Intraoperative radiation therapy
POI: Postoperative ileus
PD: Pancreaticoduodenectomy
DP: Distal pancreatectomy
LG: Local gastrectomy
TG: Total gastrectomy
PG: Proximal gastrectomy
DG: Distal gastrectomy
RCT: Randomized clinical trial.

## Supplementary Materials

Supplementary Materials Supplemental 1: gastrointestinal function assessment scale. The gastrointestinal function scale included possible gastrointestinal symptoms during the perioperative period and gastric tube drainage during the postoperative recovery period which is used to assess gastrointestinal function. The total score is 120. Supplemental 2: histogram and normal distribution curve of time to flatus. (a) EA group; (b) SEA group. Histogram and normal distribution curve showed that the postoperative flatus time data was of approximately normal distribution. Supplemental 3: histogram and normal distribution curve of time to defecation. (a) EA group; (b) SEA group. Histogram and normal distribution curve showed that the postoperative defecation time data was of approximately normal distribution. Supplemental 4: the kurtosis and skewness values of the data are between ±1 which demonstrate the data is of approximately normal distribution. .
