# Supplementary material for: Perioperative Electroacupuncture Can Accelerate the Recovery of Gastrointestinal Function in Cancer Patients Undergoing Pancreatectomy or Gastrectomy: A Randomized Controlled Trial
Source: Evid Based Complement Alternat Med. 2021 Mar 31;2021:5594263. doi: 10.1155/2021/5594263 (PMC8026294; doi:10.1155/2021/5594263)
Supplement: Supplementary Materials — Supplemental 1: gastrointestinal function assessment scale. The gastrointestinal function scale included possible gastrointestinal symptoms during the perioperative period and gastric tube drainage during the postoperative recovery period which is used to assess gastrointestinal function. The total score is 120. Supplemental 2: histogram and normal distribution curve of time to flatus. (a) EA group; (b) SEA group. Histogram and normal distribution curve showed that the postoperative flatus time data was of approximately normal distribution. Supplemental 3: histogram and normal distribution curve of time to defecation. (a) EA group; (b) SEA group. Histogram and normal distribution curve showed that the postoperative defecation time data was of approximately normal distribution. Supplemental 4: the kurtosis and skewness values of the data are between ±1 which demonstrate the data is of approximately normal distribution. . [file 5594263.f1.zip › 5594263.f1/supplemental 1. Gastrointestinal function assessment scale.docx]

Supplemental 1. Gastrointestinal function assessment scale

| Index | Standards | Score |
| --- | --- | --- |
| Flatus | No | 0 |
|  | Yes | 10 |
| Defecation | No | 0 |
|  | Yes | 10 |
| Gastric tube | Volume of drainage ≥ 4000 mL | 0 |
|  | Volume of drainage ≥ 2000 mL | 2 |
|  | Volume of drainage ≥ 1000 mL | 4 |
|  | Volume of drainage ≥ 500 mL | 6 |
|  | Volume of drainage < 500 mL | 8 |
|  | Removed | 10 |
| Intestinal feeding tube | Carried | 0 |
|  | Removed | 10 |
| Borborygmus | No | 0 |
|  | ≤ 3 times | 5 |
|  | Normal (4 or 5 times) | 10 |
| Diet | No | 0 |
|  | Water only | 3 |
|  | Liquid diet | 6 |
|  | Semi-liquid diet | 9 |
|  | Normal | 12 |
| Stomachache | Always | 0 |
|  | Usually | 5 |
|  | Rarely | 10 |
| Nausea | Always | 0 |
|  | Usually | 5 |
|  | Rarely | 10 |
| Vomiting | ≥ 3 times a day | 0 |
|  | 1 or 2 times a day | 5 |
|  | No | 10 |
| Appetite | No | 0 |
|  | Slight | 5 |
|  | Intense | 10 |
| Diarrhea | No | 0 |
|  | Yes | 3 |
| Anti-nausea drugs | Yes | 0 |
|  | No | 5 |
| Laxative/anti-diarrheal drugs | Laxative drugs | 0 |
|  | No laxative drugs or anti-diarrheal drugs | 5 |
| Gastrointestinal motility drug | Yes | 0 |
|  | No | 5 |
| Total |  | 120 |
