# Supplementary figures and images for: Perioperative Electroacupuncture Can Accelerate the Recovery of Gastrointestinal Function in Cancer Patients Undergoing Pancreatectomy or Gastrectomy: A Randomized Controlled Trial
Source: Evid Based Complement Alternat Med. 2021 Mar 31;2021:5594263. doi: 10.1155/2021/5594263 (PMC8026294; doi:10.1155/2021/5594263)

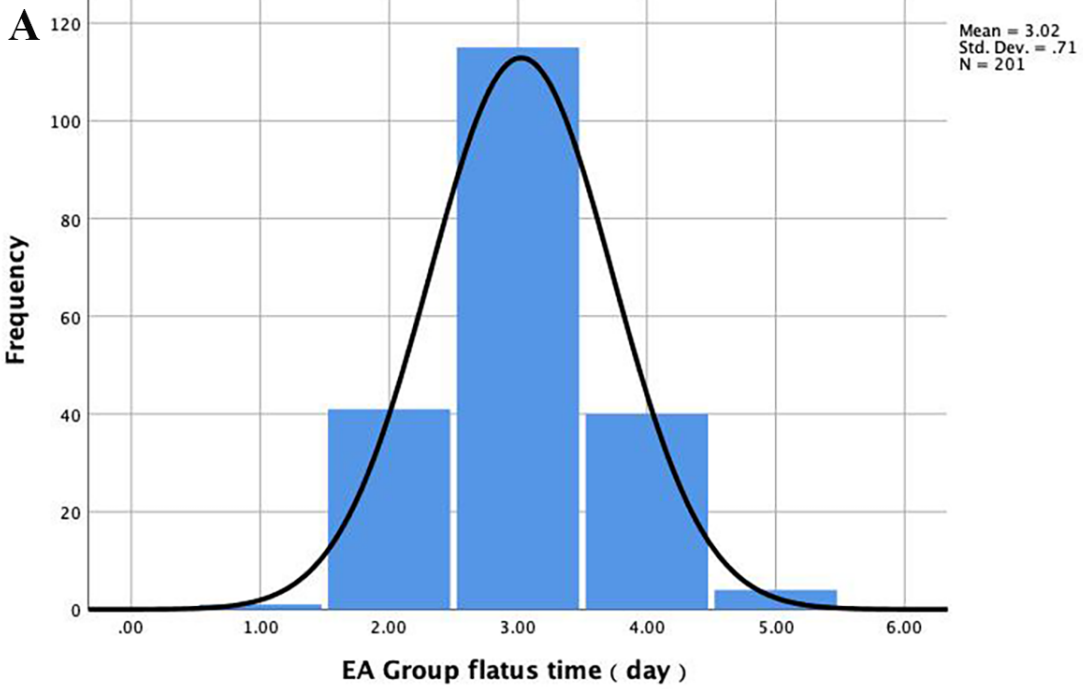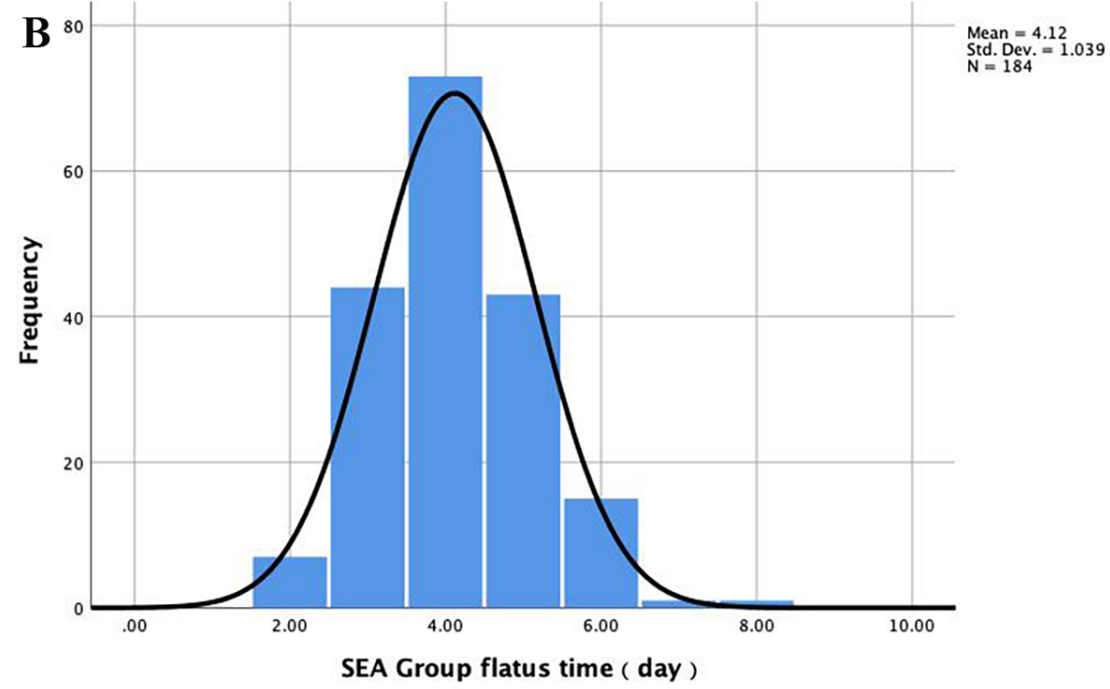

Supplement: Supplementary Materials — Supplemental 1: gastrointestinal function assessment scale. The gastrointestinal function scale included possible gastrointestinal symptoms during the perioperative period and gastric tube drainage during the postoperative recovery period which is used to assess gastrointestinal function. The total score is 120. Supplemental 2: histogram and normal distribution curve of time to flatus. (a) EA group; (b) SEA group. Histogram and normal distribution curve showed that the postoperative flatus time data was of approximately normal distribution. Supplemental 3: histogram and normal distribution curve of time to defecation. (a) EA group; (b) SEA group. Histogram and normal distribution curve showed that the postoperative defecation time data was of approximately normal distribution. Supplemental 4: the kurtosis and skewness values of the data are between ±1 which demonstrate the data is of approximately normal distribution. . [file 5594263.f1.zip › 5594263.f1/supplemental 2. Histogram and normal distribution curve of time to flatus.pdf]

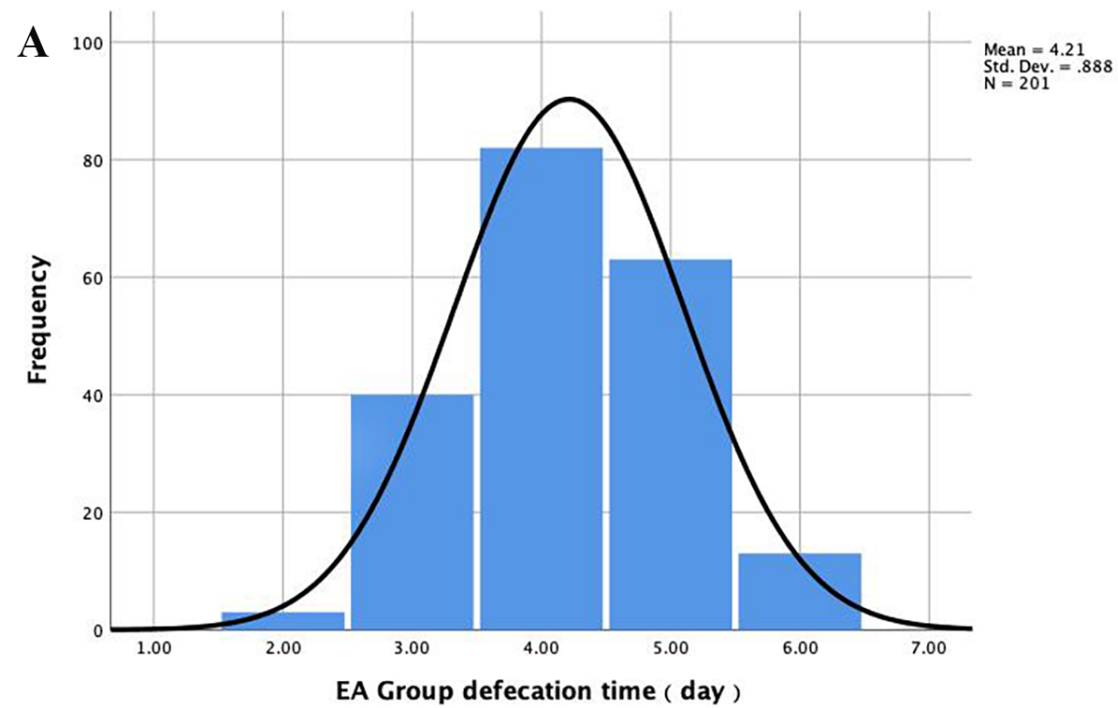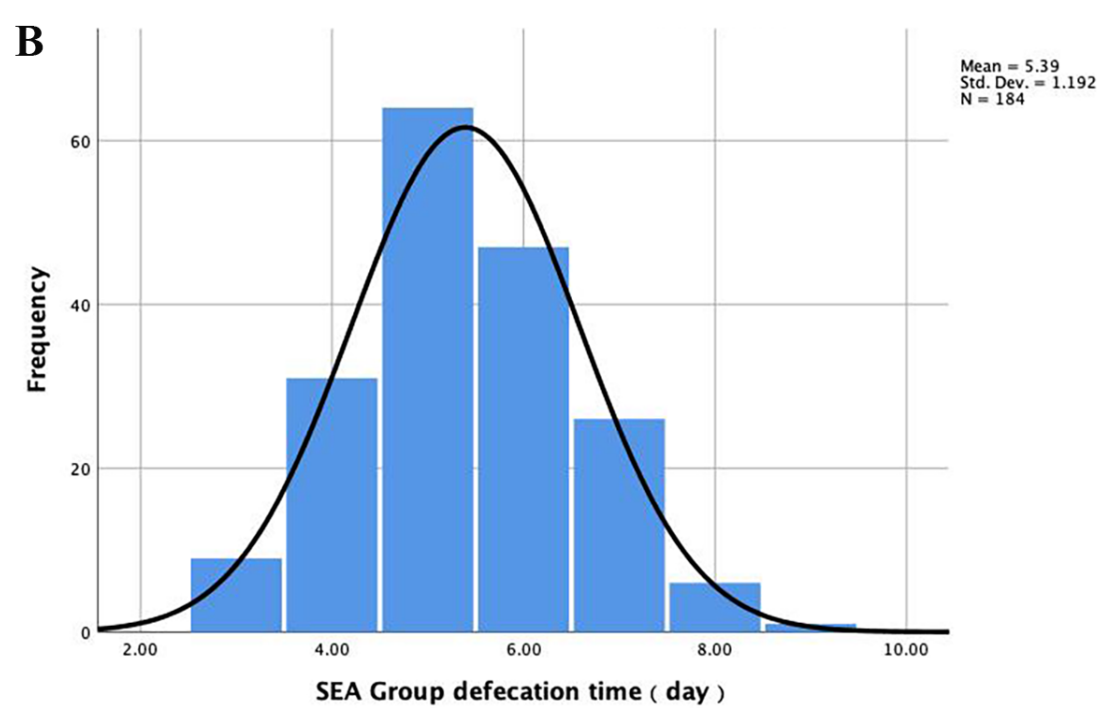

Supplement: Supplementary Materials — Supplemental 1: gastrointestinal function assessment scale. The gastrointestinal function scale included possible gastrointestinal symptoms during the perioperative period and gastric tube drainage during the postoperative recovery period which is used to assess gastrointestinal function. The total score is 120. Supplemental 2: histogram and normal distribution curve of time to flatus. (a) EA group; (b) SEA group. Histogram and normal distribution curve showed that the postoperative flatus time data was of approximately normal distribution. Supplemental 3: histogram and normal distribution curve of time to defecation. (a) EA group; (b) SEA group. Histogram and normal distribution curve showed that the postoperative defecation time data was of approximately normal distribution. Supplemental 4: the kurtosis and skewness values of the data are between ±1 which demonstrate the data is of approximately normal distribution. . [file 5594263.f1.zip › 5594263.f1/supplemental 3. Histogram and normal distribution curve of time to defecation.pdf]
